# Ictal and interictal brain activation in episodic migraine: Neural basis for extent of allodynia

**DOI:** 10.1371/journal.pone.0244320

**Published:** 2021-01-04

**Authors:** Nasim Maleki, Edina Szabo, Lino Becerra, Eric Moulton, Steven J. Scrivani, Rami Burstein, David Borsook

**Affiliations:** 1 Center for Pain and the Brain (PAIN Research Group), Boston, MA, United States of America; 2 Department of Anesthesiology, Critical Care and Pain Medicine, Boston Children’s Hospital, Harvard Medical School, Boston, MA, United States of America; 3 Department of Ophthalmology, Boston Children's Hospital, Harvard Medical School, Boston, MA, United States of America; 4 Department of Diagnostic Sciences, Craniofacial Pain Center, Tufts University School of Dental Medicine, Boston, MA, United States of America; 5 Department of Public Health and Community Medicine, Pain Research, Education and Policy Program, Tufts University School of Medicine, Boston, MA, United States of America; 6 Department of Anesthesia and Critical Care, Beth Israel Deaconess Medical Center, Harvard Medical School, Boston, MA, United States of America; 7 Department of Psychiatry, Brain Imaging Center, McLean Hospital, Harvard Medical School, Belmont, MA, United States of America; University Medical Center Goettingen, GERMANY

## Abstract

In some patients, migraine attacks are associated with symptoms of allodynia which can be localized (cephalic) or generalized (extracephalic). Using functional neuroimaging and cutaneous thermal stimulation, we aimed to investigate the differences in brain activation of patients with episodic migraine (n = 19) based on their allodynic status defined by changes between ictal and interictal pain tolerance threshold for each subject at the time of imaging. In this prospective imaging study, differences were found in brain activity between the ictal and interictal visits in the brainstem/pons, thalamus, insula, cerebellum and cingulate cortex. Significant differences were also observed in the pattern of activation along the trigeminal pathway to noxious heat stimuli in no allodynia vs. generalized allodynia in the thalamus and the trigeminal nucleus but there were no activation differences in the trigeminal ganglion. The functional magnetic resonance imaging (fMRI) findings provide direct evidence for the view that in migraine patients who are allodynic during the ictal phase of their attacks, the spinal trigeminal nucleus and posterior thalamus become hyper-responsive (sensitized)–to the extent that they mediate cephalic and extracephalic allodynia, respectively. In addition, descending analgesic systems seem as “switched off” in generalized allodynia.

## 1. Introduction

During a migraine attack, most patients develop cutaneous allodynia, defined as pain perception evoked by normally non-noxious stimuli [1, 2]. That is, patients with migraine may experience pain from common daily activities, such as wearing glasses, or taking a shower [2, 3]. The pain in migraine is thought to contain an early activation and sensitization of first-order neurons in the trigeminal ganglion characterized by limited or no skin sensitivity to the head (no allodynia), causing activation in second-order neurons in the spinal trigeminal nucleus. Whereas sensitization of the first-order neurons explains the throbbing perception of migraine headaches and their aggravation by physical activity, second-order neurons in the spinal trigeminal nucleus appear to be responsible for mediating the development of allodynia on the ipsilateral side of head (localized or cephalic allodynia), and subsequent sensitization of third-order thalamic trigeminovascular neurons (particularly in the pulvinar region of the thalamus) has been related to the development of allodynia on the contralateral side of the head (localized or cephalic allodynia) and body (generalized or extracephalic allodynia) [4].

The associated manifestations of allodynia have clinical implications including (1) period of time for effective treatment in episodic migraine (in patients with allodynia, the effectiveness of triptans is much higher when they are administered early in the attack) [1, 5], (2) differences in triggers in allodynic vs. non-allodynic migraine (patients with allodynia have more triggers than those with no allodynia) [6], and (3) predicting migraine progression (allodynia is a risk factor for migraine chronification) [7].

Most imaging research has been done between migraine attacks (i.e., interictally) [8], there are fewer studies focusing on patients during an acute migraine attack (i.e., ictally) [9]. Previous findings have shown changes in the trigeminal system in healthy subjects after noxious stimulation [10] and in patients with migraine [11]. Nevertheless, there are no studies investigating the differences between allodynic states in the pathway. Furthermore, generalized allodynia may require other brain systems (i.e., cognitive, sensory) which could provide a basis for differences in underlying brain mechanisms that may relate to treatment resistance or migraine progression [7, 12]. Altered brain systems in migraine hyperexcitability have been well-documented but the issue of how brain responses may relate to the extent of allodynia has not been assessed in imaging studies before.

Our aim was to investigate brain activation in allodynic and non-allodynic migraine patients during their ictal vs. interictal state to assess cortical and subcortical differences underlying the phenomenon of migraine-associated allodynia as defined according to changes between ictal and interictal pain tolerance threshold in response to thermal stimuli using quantitative sensory testing (QST). We focused on two questions: (1) Are there any differences in response to noxious thermal stimulation in the migraine brain between ictal vs. interictal states along the trigeminovascular pathway using functional magnetic resonance imaging (fMRI)?; and (2) How are the brain response changes manifested in the generalized allodynic state different from those in the non-allodynic state? The observations and insights gathered from the brain changes in allodynia may help understand if the progression from no allodynia to generalized allodynia engages brain areas involved in the sensory, emotional and other aspects of migraine and how each is involved.

## 2. Methods

### 2.1. Patient recruitment and consent

Subject recruitment and the experiment protocol were approved by the McLean Hospital Institutional Review Board and met the scientific and ethical guidelines for human pain research of the Helsinki Accord (http://ohsr.od.nih.gov/guidelines/helsinki.html) and the International Association for the Study of Pain. Subjects were recruited through participating physicians and online advertisements. After a telephone interview, participants were scheduled for a screening session during which all aspects of the study were described (including study procedures, time commitment, and the types of pain stimuli to be used). To ensure compliance with the inclusion and exclusion criteria prior to subject enrollment, a study physician performed a full medical examination, and a detailed medical history was taken from the patients (including information on the age of migraine onset, duration and frequency of the migraine attack, presence of aura, type of medication the patients used, and their history of allodynic status). Written informed consent was received from all participants by the study staff after answering any study-related questions.

### 2.2. Inclusion/exclusion criteria

The inclusion criteria were as follows: (i) episodic migraine as defined by the International Classification for Headache Disorders (ICHD-2) [13]; (ii) Beck Depression Inventory II (BDI-II; [14]) scores ≤ 25; (iii) had episodic migraine for at least three years or longer. The exclusion criteria were as follows: (i) other significant medical condition (except migraine); (ii) depression measured by BDI-II [14] with a cut-off score of ≥ 25 (implicating moderate to severe depression); (iii) on preventatives; (iv) positive on pregnancy or drug screening tests; (v) having MRI contraindications (such as having a heart pacemaker or unremovable body piercing etc.). Participants who took medications for their migraine headaches were asked to discontinue their pain medication for one dosing period before their scan sessions. They were allowed to continue taking these medications after the scan visit. Subjects were also asked to refrain from eating or drinking caffeinated beverages for 12 hours before their scan and from eating in general up to 6 hours before the scan session.

### 2.3. Study visits

The study consisted of two visits, one during the interictal period (baseline scan) and one during an ictal event (migraine scan). The initial interictal visit was scheduled when the patients had been migraine free for 72 hours and did not have any symptoms or signs of developing a migraine attack. For the ictal visit, arrangements were made so that the research team was available between 6am to 8pm on weekdays and weekends to scan the patients within 2.5 to 5 hours of the migraine attack onset.

### 2.4. Quantitative sensory testing (QST)

In order to determine individual sensitivity and pain thresholds to thermal stimuli, each subject participated in QST prior to each scan session. Thermal pain thresholds were measured on the face and dorsum of the hand, and the side of the body was determined based on which side the pain is felt more often during a migraine attack. The same area on the back of the hand was tested for all patients. Participants received between 3–5 thermal stimuli and the average of these values +1˚C was the threshold temperature that the participant would receive during the scan.

### 2.5. Procedure

The experimental paradigm included two visits to the McLean Hospital Brain Imaging Center. The scans during both visits consisted of a series of anatomical scans followed by a functional imaging scan. For both functional scans, a painful heat stimulus (threshold +1°C) was applied to the hand. After the ictal scan, patients were allowed to take their pain medications.

### 2.6. Stimulation paradigm

Patients rated their pain while lying in the MRI scanner during each functional scan using a rating dial. They were instructed to assess the pain intensity evoked from each stimulus using a visual analog scale (VAS; 0 = no pain, 10 = maximum pain).

### 2.7. Thermal stimulation

A Federal Drug Administration-approved Thermal Sensory Analyzer (TSA-II, Medoc Advanced Medical Systems, Ramat Yishai, Israel) was used to deliver a thermal stimulus through a probe that was adapted to rest on the hand. The probe is 1.6 x 1.6 cm, or about one-half the size of the thumb. For warm heat, stimuli were given at 41˚C on the hand (a standard temperature probe that is commonly used as a low-innocuous stimulus in studies of episodic migraine; e.g., [15–18]). For painful heat, stimuli were given at +1˚C above the subject’s individualized pain threshold value (as determined by QST) on the hand. Stimuli were repeated three times, each for a length of 25 seconds and separated by 30 seconds of no stimuli.

### 2.8. Imaging

#### 2.8.1. Image acquisition

Imaging was performed in a 3.0 T Siemens (Erlangen, Germany) Trio scanner using a circularly polarized (CP) coil. A Gradient Echo (GE) echo planar imaging (EPI) sequence (TR = 2500 ms, TE = 30 ms, resolution = 3.5 x 3.5 x 3.5 mm^3^, matrix = 64 x 64, 74 volumes, 41 slices) was used to acquire the functional images. High resolution, T_1_-weighted structural data were also collected from each patient using a 3D MPRAGE pulse sequence (TR = 2100 ms, TE = 2.74 ms, flip angle = 12°, 128 sagittal slices, resolution = 1.33 × 1.0 × 1.0 mm^3^).

#### 2.8.2. Image analysis

Functional imaging data processing and analyses were carried out using FMRIB Software Library (FSL) (www.fmrib.ax.ac.uk/fsl), version 4.1.9. The preprocessing steps included skull stripping using the Brain Extraction Tool (BET) with bias field correction and neck removal. The volumes were motion corrected using FMRIB's Linear Image Registration Tool (MCFLIRT) and B0 unwarping was performed using FMRIB's Utility for Geometrically Unwarping EPIs (FUGUE) to correct for some field inhomogeneities. The data were spatially smoothed with a Gaussian kernel of 5 mm full width at half maximum (FWHM) and a high-pass temporal filter with cutoff of 60s was applied. First-level analysis on functional imaging data for each subject was carried out using FMRI Expert Analysis Tool (FEAT) Version 5.98. The recorded temperature traces for each participant were entered as the explanatory variables (EVs) for heat stimuli. EVs were convolved with a gamma-variate hemodynamic response function. Functional volumes were spatially normalized to the Montreal Neurological Institute (MNI152) template for group analysis, and individual data of right-sided migraine patients (who were stimulated on the right side) were flipped along the y-axis so that they correspond with the patients with left-sided migraine. Thus, in the group results the left side is the ipsilateral stimulation side, while the right side is the contralateral stimulation side. Patients with no dominant migraine side were also stimulated on the left. Contrasts were defined for the non-noxious and noxious thermal stimuli for each subject, and in the group level analysis contrasts were generated to compare allodynic patients to non-allodynic patients, and to investigate patients during their ictal and interictal state. Higher level group contrast analysis was carried out to calculate the intragroup and intergroup comparison activation maps using FMRIB’s Local Analysis of Mixed Effects (FLAME1) [19]. All statistical parametric maps were thresholded using a Gaussian Mixture Model (GMM) technique [20] which is a generalized false discovery rate (FDR) correction for multiple comparisons (*p* < 0.05).

#### 2.8.3. Region of interest (ROI) analyses

In addition to the whole-brain approach, two region of interest (ROI) analyses were conducted: (1) ROIs were determined for the trigeminal ganglion and trigeminal nucleus based upon previously implicated areas in the trigeminal nociceptive pathway [21–23], and the thalamus in the standard MNI space using the MNI structural atlas (available in FSL) [24]; (2) ROIs were defined in the thalamus based on a probabilistic atlas of thalamic sub-regions segmented according to their white-matter connectivity to cortical areas [25, 26]. More specifically, the Oxford thalamic connectivity atlas was used (included in FSL), which reports probabilistic connectivity values based on diffusion tractography. This comprises bilateral thalamic subregions that have been identified to show specific anatomical connections to different cortices. For both ROI analyses, the group average contrast of parameter estimates (COPE) values were converted to percentage (%) signal change in ictal vs. interictal scans for noxious stimulation using FSL’s Featquery for which the contrast maps were thresholded according to the GMM determined thresholds.

## 3. Results

### 3.1. Patients' characteristics

**Fig 1:**
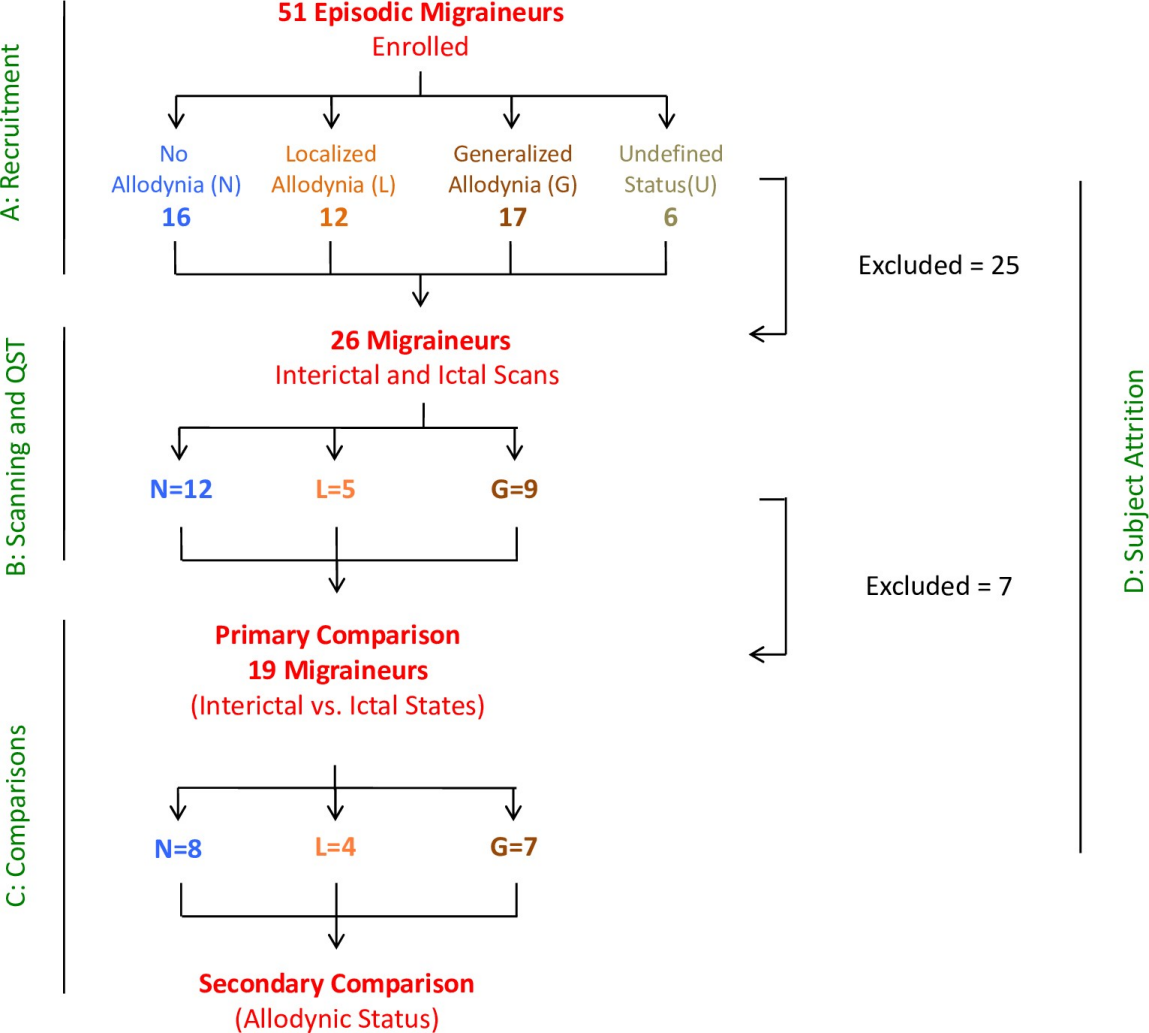
Flowchart showing the sequence of patient inclusion.

**Fig 1** shows the flowchart of patient inclusions in the study. From the 51 episodic migraine patients, 26 patients completed both scan visits for this study. However, six patients were excluded from the analysis due to the long time separation between the interictal scan and the scan obtained during the migraine attack (> 1 year). Twenty patients completed both visits within 9 months (13 females, 7 males; 44.6 ± 11.7 years old, age range: 24–61 years). One female subject was excluded later from the imaging analysis due to the concerns of residual effects of a beta-blocker. Nineteen episodic migraine patients (12 females) were included in the final imaging analysis. A detailed description of demographic and migraine attack characteristics is shown in **Table 1**. There were no significant differences between males and females in age and migraine characteristics, but females used more preventative medications than males. In addition, age, sex, migraine characteristics and medication history were not significantly different between the allodynia groups defined by QST testing (see below). All of the participants had migraine for at least more than four years and experienced five to six migraine attacks on average each month with a range of one to twelve migraine attacks per month.

**Table 1:** Demographic and clinical characteristics of the migraine patients.

**Subject group**	Female	Male	*p* value	Generalized allodynia	Localized allodynia	No allodynia	Total	*p* value
	*n* = 12	*n* = 7					(*N* = 19)	
				*n* = 7	*n* = 4	*n* = 8		
**Age**	41.3 ±12.4	44 ±12	0.65	42.4 ±13.1	43.5 ±11.9	41.6 ±12.7	19	0.97
**(years)**	(24–57)	(28–61)		(27–61)	(28–57)	(24–57)		
**Sex (female/male)**	–	–	–	5/2	2/2	5/3	19	0.84
**Age at migraine**	18.9 ±6.2	29.1 ±18	0.11	27.9 ±17.2	20.3 ±8.8	19.4 ±9.2	18	0.12
**onset (years)**	(10–33)	(8–55)		(10–55)	(14–33)	(8–32)		
**Duration (hours)**	24.3 ±23.9	19.4 ±19.7	0.66	7.6 ±6.6	21.2 ±24.1	34.3 ±23.9	14	0.49
	(4–72)	(1–48)		(1–18)	(1.5–48)	(2–72)		
**Frequency per month**	5.5 ±4	6.3 ±4.3	0.71	6.6 ±4.1	3.6 ± 3.2	6.3 ±4.3	18	0.81
	(1.5–12)	(1–12)		(1.5–12)	(1–8)	(2–12)		
**Abortive**	75%	29%	0.07	57%	50%	25%	19	0.49
**Preventative**	58%	0%	0.02*	43%	25%	38%	19	0.85
**Analgesic**	58%	100%	0.11	57%	100%	75%	19	0.28
**Aura**	3	2	0.97	2	1	2	16	0.98

Data are expressed as mean ± *SD* (range) or number of patients (%). Demographic variables (sex and age), migraine characteristics (migraine onset, duration and frequency of the migraine attack, presence of aura) and medication types that the patients were taking for their migraine attacks are shown in different groups. Preventative medications can prevent migraine attacks before they occur, abortive medications might stop migraine progression, and analgesics can relieve the pain for a few hours (but they are not intended to terminate migraine headaches). The *p* values are based on chi-square (χ2) test, Fisher's exact test, and Fisher-Freeman-Halton exact test for categorical data, and independent-sample *t*-test or analysis of variance (ANOVA) for continuous data.

* *p*< 0.05.

### 3.2. Timing of the scan visits

Time elapsed between the first and second visit ranged from 1 month to 9 months with an average of 3 months (*SD* = 2.5). Since we were interested in assessing the differences in brain activation among migraine patients based on their allodynic status defined by changes between ictal and interictal pain tolerance threshold at the time of imaging, migraine scan was delayed relative to the time of onset for an average of 4 hours [1]. Once the patients were at the imaging center at McLean Hospital the scans happened shortly after they were admitted within 34.5 minutes on average (*SD* = 19.9). During this time QST was performed to measure the pain thresholds from which the allodynic status was determined (see below). Patients were experiencing comparable levels of pain (*M* = 6, *SD* = 1, on a scale of 0 (no pain) to 10 (maximum imaginable pain)).

### 3.3. Medication history

Subjects were on a wide range of medications, including over the counter medicines (e.g., Advil, Excedrin) and prescribed medications (e.g., Imitrex, Maxalt). **S1 Table in S1 File** lists the medication history for each participant in the study. While a number of patients had a history of being on preventative medication, none of them were currently on such medication (except for one subject that was later excluded from the analysis). Subjects abstained from taking their migraine medications for one dosing interval prior to their interictal scan session. They also had to refrain from taking their medication on the day of their ictal visit until after the scan was completed. Medication did not change between the first and second visit.

### 3.4. Pain tolerance threshold and allodynia state

**Fig 2:**
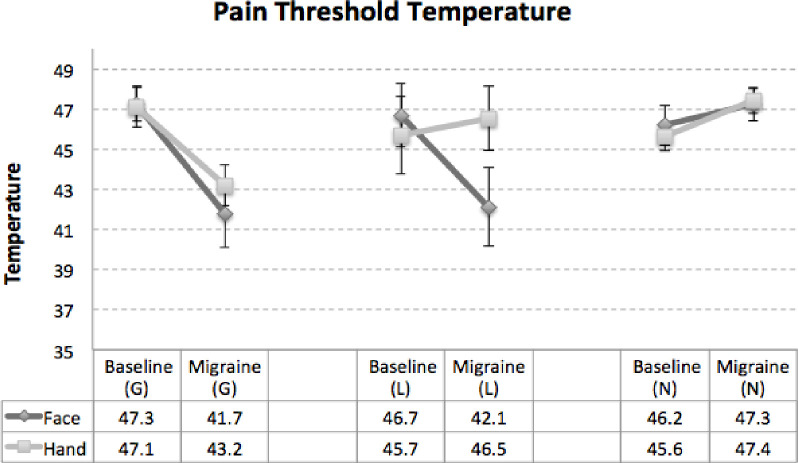
Change in the pain tolerance thresholds. Allodynia state was determined according to the changes in the pain tolerance thresholds between interictal and ictal visits on face and hand. If the pain tolerance change was >3°C on both face and hand the patient was categorized as having generalized allodynia. Localized allodynia was defined if >3°C change was observed on face but not hand. If there was no change or if the change was < 3°C on face and hand that was considered no allodynia. In total there were 7 patients who experienced generalized allodynia and 8 who were categorized as having no allodynia with the remaining 4 experiencing localized allodynia.

Pain tolerance thresholds were determined on the hand and the face for all of the patients during both interictal (baseline (B)) and ictal (migraine (M)) visits using QST. The results for the allodynia status are presented in **Fig 2**. Allodynia state was determined based on the changes in the pain tolerance thresholds (>3°C) between face and hand when stimulated by the heat [1, 27, 28]. In total there were seven patients who experienced no allodynia (Face: 46.2°C (B) vs. 47.3°C (M), Hand: 45.6°C (B) vs. 47.4°C (M)), four patients who were categorized as having localized allodynia (Face: 46.7°C (B) vs. 42.1°C (M), Hand: 45.7°C (B) vs. 46.5°C (M)) and the remaining eight patients experienced generalized allodynia (Face: 47.3°C (B) vs. 41.7°C (M), Hand: 47.1°C (B) vs. 43.2°C (M)).

### 3.5. Visual analog scale (VAS) rating of pain intensity

The noxious heat stimulus (threshold +1°C) applied to the dorsum of the hand evoked on average a pain intensity of 6.1 ± 3 (SD) during the interictal scans and 7.4 ± 2.6 (SD) during the ictal scans on the 0–10 scale. In spite of the fact that participants were pain-free in the baseline (B) visit and experiencing head pain in the migraine (M) visit, there was no significant difference between the VAS ratings between the ictal vs. interictal visits (*t*(18) = 1.84, *p* = 0.1). The VAS data were compared within the allodynia groups as well and no significant differences were found between the ictal vs. interictal VAS scores in the allodynic groups: no allodynia (Face: 6.6 (B) vs. 6.3 (M), Hand: 5.4 (B), vs. 7.1 (M)), localized allodynia (Face: 4.6 (B) vs. 6 (M), Hand: 4.9 (B) vs. 6(M)), generalized allodynia (Face: 7.34 (B) vs. 7.1 (M), Hand: 7.9 (B) vs. 7.2 (M)).

### 3.6. Imaging results

**Fig 3:**
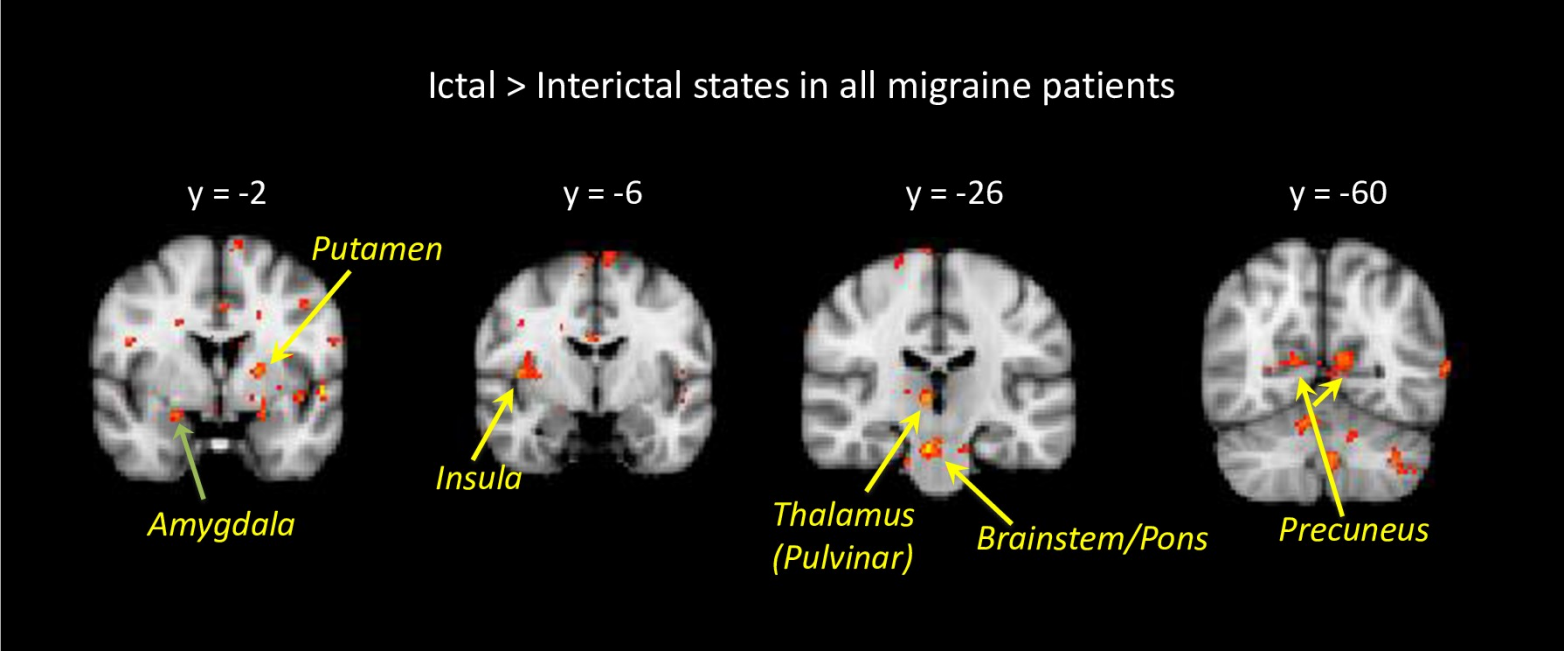
Brain activation for ictal vs. interictal state. Contrast analysis of the ictal visit vs. interictal visit in response to noxious thermal stimulation on the left hand is presented. Hot colors (yellow to red colors) indicate stronger activation during the ictal state. The results are based on longitudinal data of 19 migraine patients, and non-noxious heat vs. noxious heat contrast maps. Statistical maps were thresholded and corrected for multiple comparisons using Gaussian mixture modeling (GMM) approach. Coordinates are in Montreal Neurological Institute (MNI) space.

**Fig 4:**
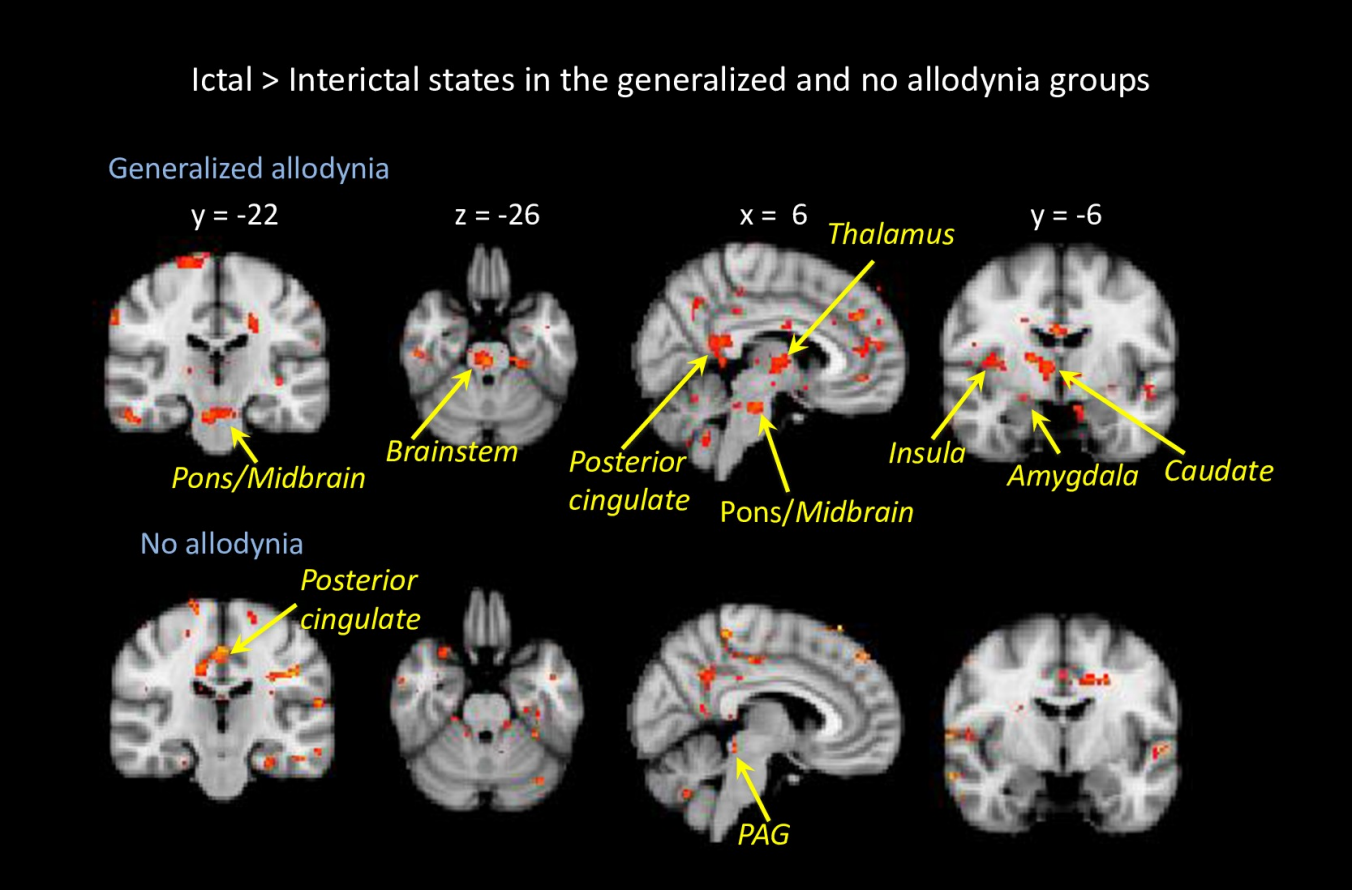
Brain activation in generalized allodynia vs. no allodynia. Contrast analysis of the ictal vs. interictal visits in response to noxious thermal stimulation on the left hand is presented for patients with generalized allodynia vs. patients with no allodynia. Hot colors (yellow to red colors) indicate stronger activation during the ictal state. The results are based on longitudinal data of 7 patients with generalized allodynia and 8 patients with no allodynia. Statistical maps were thresholded and corrected for multiple comparisons using Gaussian mixture modeling (GMM) approach. Coordinates are in Montreal Neurological Institute (MNI) space.

**Fig 5:**
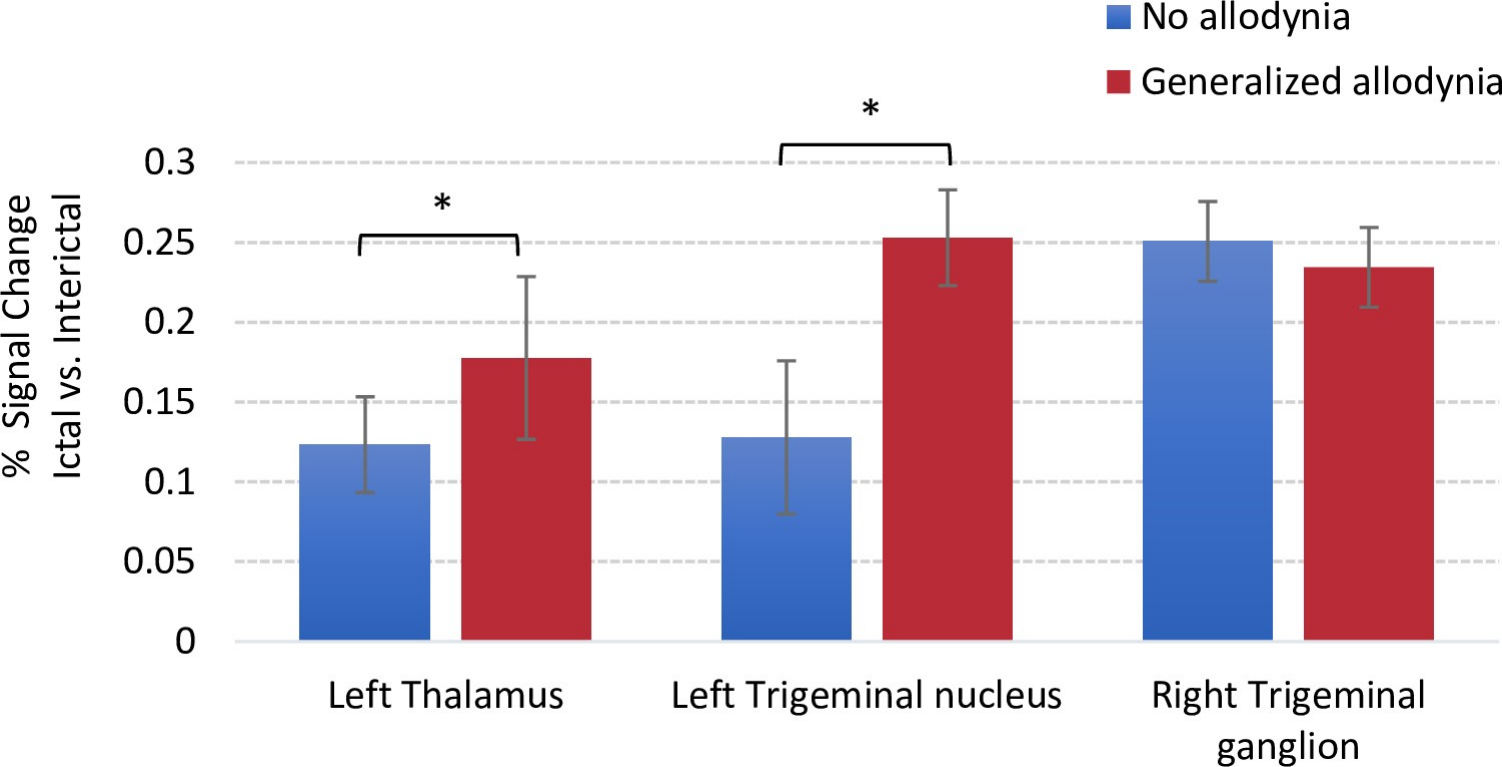
Signal change differences along the trigeminal pathway based on Region of Interest (ROI) analysis. Signal change values were extracted using Featquery. For those patients who received thermal stimulation on the right side, the brains were flipped along the y-axis so that all images were in accordance regarding the site of the migraine (and the stimulation side). The left side is the ipsilateral stimulation side and the right is the contralateral stimulation side. The group average results for percentage signal change between generalized and no allodynia groups are shown for the left thalamus and left trigeminal nucleus ipsilateral to the side of stimulation. For the trigeminal ganglion, the signal change is reported for the contralateral region (right side). Error bars represent standard deviations. The * symbol indicates group differences at a significant level of p < 0.05.

**Fig 6:**
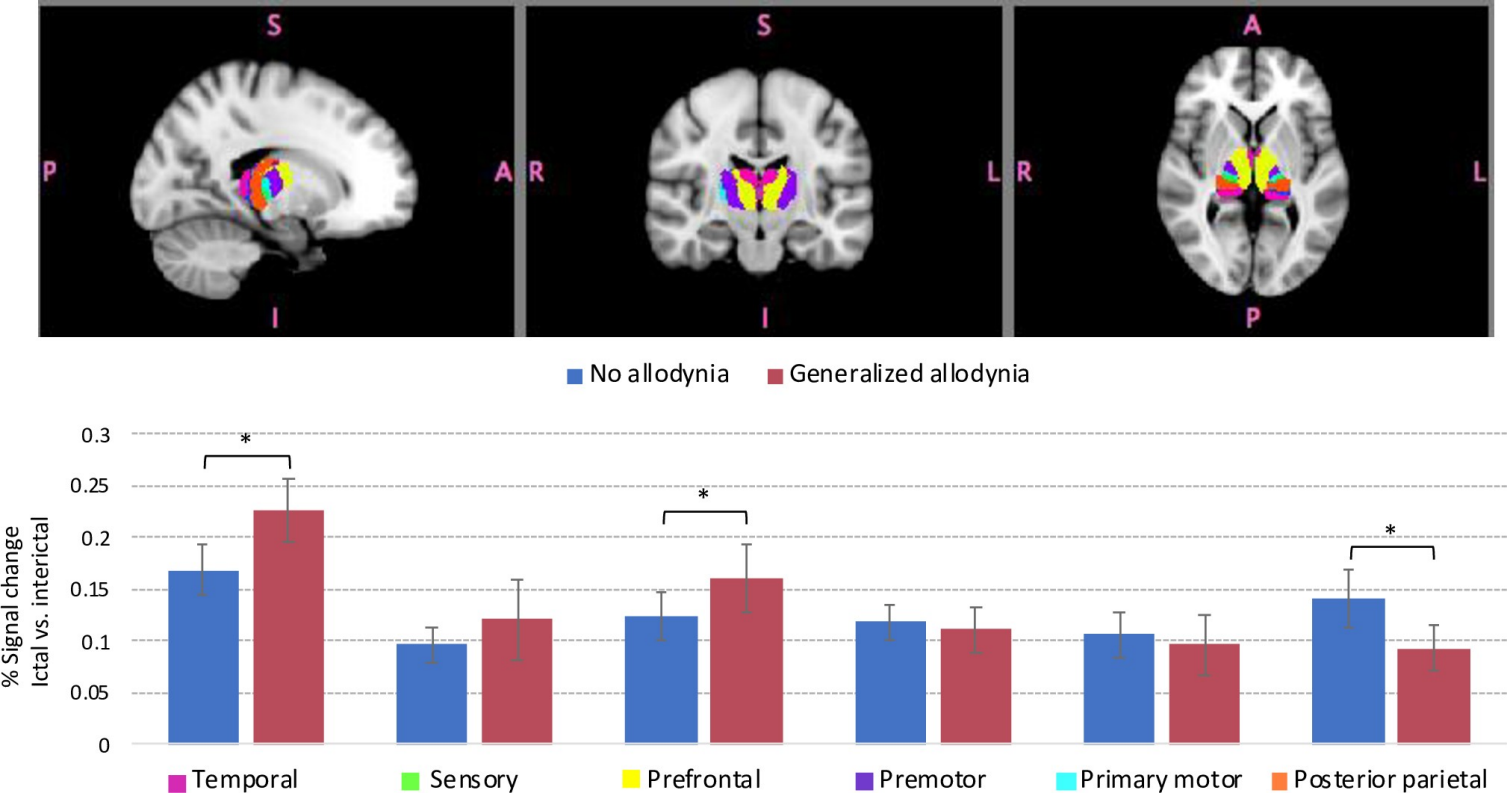
Region of Interest (ROI) based comparison results for percentage (%) signal change in various thalamic regions according to the cortical connectivity in ictal vs. interictal scans. Signal change values were extracted using Featquery. Thalamic sub-regions (indicated by different colors) were segmented based on the white-matter connectivity between the thalamus and cortex. The label for each color is listed in the x-axis. Bars represent changes in the percentage of the average signal change between ictal vs. interictal activation in response to noxious heat applied to the dorsum of the hand for patients with generalized allodynia and with no allodynia. The main differences are in the thalamic regions that have connections with temporal and prefrontal cortical regions with an increased signal change in generalzied allodynia patients. In contrast, thalamic regions with connectivity to the posterior parietal regions showed stronger signal change in the no allodynia group. Error bars represent standard deviations. The * symbol indicates group differences at a significant level of p < 0.05.

Imaging results are presented in **Figs 3** to **6**. Group average findings for brain activation in response to noxious heat (threshold +1°C) applied to the dorsum of the hand during the interictal and ictal scans are presented in **S2A** and **S2B Tables in S1 File**.

#### 3.6.1. Interictal (baseline) state

Ipsilateral activation in response to noxious heat stimulation was observed in the thalamus, middle cingulate, precentral, and superior frontal gyrus. Contralateral activation was found in the anterior insula, caudate, palladium, temporal pole, middle temporal, and postcentral gyrus. And bilateral activation was seen in response to stimulation in the supramarginal, inferior parietal, inferior operculum gyrus and cerebellum, although activation in the cerebellum was mainly contralateral.

#### 3.6.2. Ictal (migraine) state

Ipsilateral activation in response to noxious heat stimulation was observed in the middle cingulate, precentral, and superior frontal gyrus. Contralateral activation was seen in the pons, anterior insula, caudate, palladium, temporal pole, middle temporal, and postcentral gyrus, as well as in the cerebellum. Bilateral activation was observed in thalamus, rolandic operculum in the occipital cortex, supramarginal, inferior parietal and inferior operculum gyrus.

#### 3.6.3. Ictal vs. interictal states

Contrast analysis results for brain activation in ictal vs. interictal scans in response to noxious heat (threshold +1°C) applied to the hand are shown in **Fig 3** and the corresponding activations are shown in **Table 2A** and **2B**. The main differences were significantly stronger ipsilateral activation during the migraine scan in the anterior and posterior insula, supramarginal gyrus and cerebellum, as well as stronger contralateral activation in the anterior and middle cingulate, trigeminal nucleus and brainstem (pons). Significantly stronger bilateral activation during the migraine attack was found in the occipital cortex, and posterior cingulate. There were also brain regions that showed increased activation during the interictal scan compared to the ictal scan. The main differences were in the frontal cortical regions and temporal regions. There was an increased activation in the ipsilateral precentral gyrus, postcentral gyrus, as well as putamen. Contralateral stronger responses were found in the inferior, superior and middle temporal gyrus.

**Table 2:** a. Whole brain contrast results in ictal vs. interictal scans (ictal > interictal). b. Whole brain contrast results in ictal vs. interictal scans (interictal > ictal).

Regions	Side	z-stat	Peak MNI coordinates			
			X(mm)	Y(mm)	Z(mm)	Vol(cm^3^)
Frontal						
Superior_Orbital	R	3.3814	20	42	38	0.368
Superior_Orbital	R	2.8114	18	28	58	0.344
Supp_Motor_Area	L	2.7844	-8	-2	72	0.32
Parietal						
SupraMarginal	L	2.6074	-58	-46	28	0.232
Occipital						
Rolandic_Operculum	R	2.6647	44	-4	8	0.32
Calcarine	L	2.8037	-12	-52	10	0.232
Calcarine	L	2.4985	-8	-62	12	0.504
Temporal						
Heschl	R	2.6146	40	-28	14	0.232
Cingulum						
Anterior	R	2.6638	12	42	8	0.256
Middle	R	2.6727	16	22	30	0.256
Middle	R	2.767	2	8	38	0.36
Post	L	3.0431	-6	-42	18	0.472
Post	R	2.9978	4	-44	18	0.448
Insula						
Anterior	L	2.6433	-30	20	-12	0.232
Posterior	L	2.4625	-40	-2	-8	0.352
Brainstem / Cerebellum						
SpV	R	2.8273	4	-42	-52	0.432
Cerebellum_9	R	2.8033	6	-54	-44	0.448
Cerebellum_Crus2	L	2.3965	-38	-56	-40	0.4
Cerebellum_8	L	2.6682	-4	-60	-38	0.616
Cerebellum_Crus1	L	3.1633	-32	-68	-32	0.64
Pons	R	3.6267	4	-26	-24	0.68
**b.**						
**Regions**	**Side**	**z-stat**	**Peak MNI coordinates**			
			**X(mm)**	**Y(mm)**	**Z(mm)**	**Vol(cm**^**3**^**)**
Frontal						
Middle_Orbital	R	2.1648	34	50	20	0.32
Superior_Medial	L	2.7724	-6	38	36	0.92
Middle_Orbital	R	2.1369	34	38	26	0.224
Middle	L	3.2923	-28	22	54	0.744
Middle_Orbital	R	2.359	30	14	52	0.248
Middle	L	2.3365	-32	10	50	0.216
Middle_Orbital	R	2.6035	36	8	52	0.312
Precentral	L	3.2645	-44	2	20	0.544
Precentral	L	2.0164	-40	-24	64	0.376
Paracentral_Lobule	L	2.3157	-2	-26	60	0.248
Parietal						
Postcentral	L	2.5761	-54	-16	24	0.624
Postcentral	L	2.2658	-48	-18	56	0.264
Postcentral	L	2.6175	-46	-22	42	0.632
Inferior	L	2.6969	-38	-26	38	0.432
Occipital						
Inferior	L	2.5095	-46	-60	-14	0.432
Temporal						
Fusiform	R	2.7741	36	-6	-30	0.536
Superior	R	2.2061	48	-40	10	0.32
Inferior	R	2.7627	56	-44	-12	0.32
Superior	R	2.3231	62	-46	18	0.232
Middle	R	2.5535	48	-52	12	0.224
Middle	R	-3.0756	42	-54	12	0.464
Middle	R	-2.6396	52	-56	0	0.488
Middle	R	-2.5879	48	-64	2	1.144
Cingulum						
Middle	L	-2.3467	-12	-28	46	0.296
Insula						
Anterior	R	-2.4705	32	20	10	0.4
Sub-Cortical						
Putamen	L	-2.9633	-20	16	-2	0.408
Caudate	R	-2.3917	10	12	0	0.24
Brainstem / Cerebellum						
Cerebellum_4_5	R	-2.4113	30	-44	-20	0.216
Cerebellum_7b	R	-2.7538	34	-66	-46	0.52
Cerebellum_8	R	-2.2854	20	-70	-42	0.28

2a: R, right; L, left. Brain activation in ictal vs. interictal states (ictal > interictal) in response to noxious heat applied to the dorsum of the hand. The results are based on longitudinal data of 19 migraine patients, and noxious heat vs. non-noxious heat contrast maps. Coordinates are in Montreal Neurological Institute (MNI) space.

2b: R, right; L, left. Brain activation in ictal vs. interictal states (interictal > ictal) in response to noxious heat applied to the dorsum of the hand. The results are based on longitudinal data of 19 migraine patients, and noxious heat vs. non-noxious heat contrast maps. Coordinates are in Montreal Neurological Institute (MNI) space.

#### 3.6.4. Patients with generalized allodynia vs. no allodynia

Additional analyses were performed to compare brain activation in ictal vs. interictal scans in response to noxious heat in patients who showed changes in pain threshold in both face and hand (generalized allodynia, n = 7), and in the patients who showed no change in the pain thresholds between the two visits (no allodynia, n = 8). The results are presented in **Fig 4**. The main differences between the two groups were in the activation of the contralateral brainstem (pons), insula and thalamus, which were present only in the generalized allodynia group and not in the no allodynia group. On the other hand, the no allodynia group showed increased activity during a migraine attack in the posterior cingulate and periaqueductal gray (PAG) that was not observed in the generalized allodynia group.

In **Fig 5** ROI-based comparison results for % signal change between the interictal and ictal scans for noxious stimulation of the hand are shown. The activations are presented along the trigeminal nociceptive pathway in patients with generalzied vs. no allodynia for the left thalamus and left trigeminal nucleus, ipsilateral to the side of stimulation and for the trigeminal ganglion on the contralateral side. The results show a higher level of signal change, suggestive of an increased hypersensitivity, for generalized allodynia vs. no allodynia in the thalamus and the trigeminal nucleus. The level activity for the trigeminal ganglion was comparable between the two groups.

The results in **Fig 6** present % signal changes in ictal vs. interictal scans for thalamic sub-regions according to their cortical connectivity. For this analysis, thalamic segmentation was performed using a probabilistic atlas of sub-thalamic regions that were generated based on white-matter connectivity between the thalamus and cortical areas [25, 26]. The bars represent changes in the percentage of the average signal change between ictal vs. interictal brain activation in response to noxious heat (threshold +1°C) for subjects with generalized allodynia and subjects with no allodynia. For both groups the biggest changes were in the thalamic region with connectivity to temporal and prefrontal regions in the cortex. The main differences were found in the thalamic regions that have connections with temporal and prefrontal cortical regions with a stronger signal change in generalzied allodynia patients. In the no allodynia group, there was a stronger signal change in the posterior parietal region.

## 4. Discussion

### 4.1. Overall findings

We designed a study to assess the brain activation in migraine patients in response to noxious thermal stimulation during both a migraine attack (ictal) and between attacks (interictal) in the context of the changes in the thermal pain tolerance threshold as an index of allodynia. Aside from the allodynic measures, there are a number of features that distinguish this study from others: (1) the migraine happened naturally and was not induced [29–32]; (2) the timing of the ictal scan relative to the onset of the attack was considerably consistent and similar among the patients [4]; and (3) the ictal scan happened at a timepoint at which allodynic symptoms would be present [1, 4, 9].

### 4.2. Thermal thresholds differentiate allodynic state

Differences in thermal thresholds between ictal vs. interictal visits for migraine patients were used to categorized patients’ allodynic state which allowed defining three distinct groups of no-allodynia, localized allodynia, and generalized allodynia with distinct pain tolerance profiles as shown in **Fig 2**. Questions about experiencing allodynic symptoms were also asked before enrolling patients to ensure compliance with the inclusion criteria. QST is sometimes described as the gold standard for detecting whether a patient with migraine has allodynia at a particular time [3, 27], although it is also prone to temporal sampling bias. Nevertheless, these patterns are consistent with our prior reports on the development of allodynia in migraine patients [1, 4, 9] and were used to classify patients as shown in **Fig 1**.

### 4.3. Brain activation differences between ictal and interictal states

As shown in **Fig 3** and **Table 2A and 2B** we observed differences between these two states with increased activation to thermal stimuli in trigemino-thalamic pathways (brainstem, including the trigeminal nucleus, pons and thalamus) and other brain regions (including the cingulate, temporal, anterior and posterior insula cortices, and cerebellum). Activation in these regions to thermal stimuli was previously reported in interictal migraine patients by our group [8, 33–37] and others [38–40]. These areas seem to be enhanced during the ictal state vs. interictal state by thermal stimulation.

### 4.4. Differences between allodynic states–trigemino-thalamic pathway

Our major findings were the observed differences between the no and generalized allodynia groups including increased activations in ictal vs. interictal state of the contralateral brainstem (pons), insula and thalamus, which were observed only in the generalized allodynia group and not in the no allodynia group, and that the no allodynia group showed increased activation during a migraine attack in the posterior cingulate and periaqueductal gray (PAG) that was not observed in the generalized allodynia group.

These findings suggest that in the no allodynia group, the PAG does what it supposes to do which is inserting inhibitory effects on ascending nociceptive neurons in spinal and trigeminal dorsal horn [41, 42]. Allodynia may be spatially distributed as defined in abnormal spatial distribution of pain in fibromyalgia [43, 44]. On the other hand, larger activation in the thalamus, pons and insula of generalized allodynic patients may suggest that the development of generalized allodynia is more complicated than previously thought. The development of hyper-responsiveness in the spinal trigeminal nucleus and posterior thalamus may depend not only on the continuous arrival of pain signals from the meninges, but also on some sort of a “breakdown” in the proper function of brainstem and cortical areas that regulate thalamocortical inputs.

### 4.5. Differences between allodynic states and ictal vs. interictal phases–non-trigemino-thalamic pathway

As noted in the results, regions showing increased activation in ictal vs. interictal state outside the traditional trigemino-thalamic pathways included a number of cortical regions: the cingulate cortex, orbitofrontal regions, the anterior and posterior insula, the calcarine, and the supramarginal cortices. The cingulate displayed greater increases in the contrast for ictal vs. non-ictal state for the anterior (ACC), middle (MCC) and posterior (PCC). These regions reflect possible complex involvement in pain emotion [45] and other complex processes, such as outcome evaluation [46], and the ACC is activated in migraine using experimental heat stimuli [16, 17]. The MCC is putatively involved in pain/avoidance and reward approach functions amongst others [47]. The PCC is considered to be involved in functions that include arousal and attention [48]. The orbitofrontal cortices also showed a difference between the two states. This region is involved in emotional and executive functions [49] as well as reward value for taste [50] and may thus be implicated in alterations in both taste and smell in patients with migraine. The supramarginal cortex, a sensory association cortex is involved in spatial perception and spatial contrast which is abnormal in migraine patients during their attack [51] is also activated by thermal stimuli in the interictal period. Other studies do not shed light on functionality but confirm changes in this region–i.e., thinned cortex in the supramarginal temporal region reported in episodic migraine [52]. Some of these regions were strongly activated in the allodynic state as well during the ictal scan (compared to the interictal scan). The insula is well known to participate in migraine [34], and while the anterior component considered to be involved in autonomic changes and salience [53], the posterior insula is associated with pain related changes [54]. In the generalized allodynia group increased subcortical activations were also observed in regions (i.e., amygdala, caudate) associated with altered pain and emotional processing in migraine [37, 55–57]. Furthermore, the stronger activation of the brainstem in the allodynic state is in line with the notion that the brainstem is likely to contribute to the sensory processing alterations in migraine and might be related to the intensity of allodynia and pain hypersensitivity [15, 58].

Thus, multiple cortical and subcortical regions display significantly increased responsivity to noxious heat during the ictal (and allodynic) state. A few findings contribute to these enhanced profiles. As noted in our prior report, cortical connectivity may also be involved in cortico-cortical connections noted for sensory areas in migraine [35] suggesting integration of information across sensory modalities. The possible implication of this is that areas that are involved in sensory allodynia may contribute or be driven in part by other sensory manifestations of supersensitized primary cortical areas based on the concept of central sensitization. These increased activations suggest that normal function is in abeyance and thus multiple cortical behavioral manifestations may be present in patients with allodynia. These have not been evaluated in migraine patients.

### 4.6. Caveats

There are a number of caveats that are worth consideration: (1) *Numbers of subjects*: Getting scans of migraine patients during their migraine attack is a real challenge. Although 51 patients were recruited to the study, and our data for ictal vs. interictal comparison were reasonable (26 patients completed both visits, 19 patients were included in the final analysis), the patient numbers for comparisons for the allodynic vs. non-allodynic states were small. However, our psychophysics measures helped “phenotyping” these subgroups. Considering that this was a prospective clinical imaging study, and the first visit was the interictal scan, it might be understandable that less participants came back to the ictal visit where brain activations were measured during the migraine headache. Since we were interested in the neural responses to noxious stimulation in the ictal vs. interictal states and how the changes manifest in the generalized allodynic state are different from those in the non-allodynic state, we could only focus on patients who completed both visits. Also, no healthy controls were included in the study. (2) *Migraine cycle*: Although all interictal scans were performed at least 72 hours after the last migraine attack and the patients did not have migraine during the baseline visit, their headaches were not monitored following the interictal scan. This information might have been useful for determining the specific phase of the migraine cycle. (3) *Allodynic state*: In patients who experience mild forms of allodynia, it is likely that the symptoms of allodynia may not always be present or experienced similarly with every single attack. As such, symptoms experienced on ‘average’ by a patient (as it is also assessed by allodynia symptoms questionnaires) and the QST-based determinations at a given time may not always align. (4) *Order effects*: There is an order effect due to subjects not being randomized between interictal and ictal-phase scan sessions which was not possible to avoid in this study. (5) *Laterality*: When the patients came for their ictal scan the stimulation was applied on the side that patients were feeling the most pain, or if it was equal on both sides it was applied to the left side. For all of the subjects consistently the sites were the same for both scans. However, since for the analysis the data from the right-sided migraine patients were flipped and analyzed together with the data from the left-sided migraine patients, it is difficult to make any inferences about the laterality effects of the migraine itself in the results. Flipping the brain images could be also a limitation because of the structural and functional differences between the right and left hemispheres [59]. (6) *Medication*: The issue of medication (noted in **S1 Table in S1 File**) used by the patients is always a confound in differentiating between the disease and medication effects on the brain. The patients were asked to refrain from taking any medications for 48 hours before their scan. In this study, most patients were on either triptans or non-steroidal anti-inflammatory drugs (NSAIDs) or no medication. However, since this was a paired-wise comparison and each subject served as his/her own control, at an individual subject level the residual effects of medications were canceled out in the analysis of differences between interictal and ictal scans.

### 4.7. Conclusions

Allodynia during a migraine attack is quite common and present in over 60% of patients. Based on the present findings, there are distinct patterns and activations in the no vs. generalized allodynia patients and a potential trigeminovascular pathway was detected that may facilitate the development of generalized allodynia. In particular, it seems that increased functional activity of the spinal trigeminal nucleus and posterior thalamus during an attack distinguishes patients who are allodynic vs. those who are not during their attacks. Furthermore, the thalamic subregion that connects to the temporal and prefrontal cortices shows higher activation during a migraine attack. Previous studies have demonstrated activation of the temporal pole during an attack and changes in plasticity of the temporal cortex in patients with high migraine frequency [34, 36].

## Supporting information

S1 File (DOCX)
