# Supplementary material for: Ictal and interictal brain activation in episodic migraine: Neural basis for extent of allodynia
Source: PLoS One. 2021 Jan 4;16(1):e0244320. doi: 10.1371/journal.pone.0244320 (PMC7781392; doi:10.1371/journal.pone.0244320)
Supplement: S1 File — (DOCX) [file pone.0244320.s001.docx]

**S1 Table. Medication history.** All medications the patients had a history of taking.

| **#** | **Abortive** | **Preventative** | **Analgesic** | **Other** |
| --- | --- | --- | --- | --- |
| 1 | - | - | Ibuprofen (Advil) | Valsartan (Diovan) |
| 2 | Sumatriptan (Imitrex) | - | Excedrin | - |
| 3 | Rizatriptan (Maxalt) | - | - | - |
| 4 | Sumatriptan (Imitrex) | - | - | Fluoxetine (Prozac) |
| 5 | Zolmitriptan (Zomig) | - | Excedrin | Amitriptyline |
| 6 | Naratriptan (Amerge), Eletriptan (Relpax) | - | Indometacin (Indocin), Excedrin | - |
| 7 | Naratriptan (Amerge) | Inderal (Propanolol) | Acetaminophen (Tylenol) | - |
| 8 | Zolmitriptan (Zomig), Eletriptan (Relpax) | - | Ibuprofen (Advil) | Vivitrol (Naltexone) |
| 9* | Erogotamine/Caffeine (Cafergot) | Beta-Blocker | - | - |
| 10 | - | Ca2+ Channel Blocker | - | OnabotulinumtoxinA (Botox) |
| 11 | Caffeine (Fioricet) | Topiramate (Topamax) | - | - |
| 12 | - | - | Tylenol Migraine | - |
| 13 | - | - | Aspirin, Excedrin | - |
| 14 | - |  | Ibuprofen (Advil) |  |
| 15 | Rizatriptan (Maxalt) | - | Acetaminophen (Tylenol) | - |
| 16 | - | - | Naproxen (Aleve) | - |
| 17 | - | - | Ibuprofen (Advil) | - |
| 18 | Frovatriptan (Frova) | - | Ibuprofen (Advil) | Elavil (Amitriptyline) |
| 19 | Rizatriptan (Maxalt) | - | Midrin, Ibuprofen (Advil) | - |
| 20 | - | - | Ibuprofen (Advil) | - |

Patients abstained from these medications for one dosage cycle prior to the scan session.

*Not included in the imaging analysis

**S2a Table. Group average results during interictal phase.** Brain activations in interictal (baseline) state in response to noxious heat applied to the dorsum of the hand.

**Regions Side z-stat Peak MNI coordinates**

**X(mm) Y(mm) Z(mm) Vol(cm^3^)**

Frontal

Inferior_Triangular R 4.0321 42 22 4 3.448

Supp_Motor_Area L 3.9014 0 20 52 5.176

Superior L 3.8814 -16 18 64 1.856

Inferior_Operculum R 4.0777 54 14 18 0.872

Superior L 3.8375 -16 10 66 1.624

Inferior_Operculum R 4.5394 54 10 12 2.408

Inferior_Operculum L 4.0904 -52 10 14 7.224

Supp_Motor_Area L 4.9521 -4 8 48 10.432

Supp_Motor_Area L 3.9845 -6 8 68 1.032

Inferior_Operculum R 4.3188 54 8 24 6.28

Supp_Motor_Area R 4.1926 8 6 50 5.24

Supp_Motor_Area R 4.1879 6 6 54 1.888

Supp_Motor_Area L 4.3051 0 6 60 0.824

Middle L 3.8449 -36 6 52 3.72

Supp_Motor_Area L 4.0678 -4 4 66 1.96

Precentral L 4.3863 -48 4 34 2.528

Precentral L 4.1664 -42 0 52 0.856

Precentral L 4.4367 -40 -4 54 2.896

Superior L 4.1372 -24 -4 58 2.256

Superior L 3.943 -24 -6 52 1.392

Superior L 3.9289 -22 -6 66 1.76

Precentral L 4.5723 -34 -20 50 4.32

Precentral L 3.9625 -40 -20 64 3.336

Parietal

SupraMarginal R 4.6721 62 -24 30 4.2

SupraMarginal L 4.2328 -58 -24 26 11.272

Postcentral R 4.308 50 -24 46 1.456

SupraMarginal R 4.446 62 -30 30 1.208

Inferior R 4.6732 54 -30 50 1.968

SupraMarginal R 4.2052 48 -32 46 0.8

SupraMarginal R 4.2626 56 -34 30 1.824

SupraMarginal R 4.2909 52 -36 40 3.136

Inferior R 3.9074 50 -36 52 0.648

SupraMarginal L 3.8791 -56 -38 36 0.976

Postcentral R 3.8697 40 -38 56 4.616

Inferior L 3.9688 -50 -38 36 0.664

Inferior L 3.9843 -44 -42 56 2.16

Inferior R 4.0226 50 -44 48 1.552

Inferior L 4.0934 -30 -44 42 3.64

Inferior L 4.2787 -42 -46 46 6.608

Temporal

Pole_Superior R 4.2133 54 12 -6 1.424

Superior R 4.9407 62 -30 22 1.896

Middle R 3.8845 58 -50 8 1.528

Middle R 3.914 54 -56 10 0.912

Middle R 4.0494 54 -58 0 2.44

Fusiform L 4.4277 -42 -64 -16 5.704

Middle R 4.2903 56 -66 0 3.736

Lingual R 3.9973 8 -66 -8 2.128

Fusiform R 3.9151 44 -68 -20 4.64

Fusiform L 3.8495 -28 -68 -14 1

Cingulum

Middle L 4.0275 2 20 36 4.272

Middle L 4.0391 -2 18 38 0.704

Insula

Insula_Anterior R 3.8987 40 22 -8 3.44

Insula_Anterior R 4.7115 34 18 4 2.384

Insula_Anterior R 4.4459 42 12 -6 8.36

Insula_Anterior R 3.8746 48 12 -6 0.416

Sub-Cortical

Caudate R 3.8938 16 4 14 1.088

Pallidum R 3.8529 24 0 4 6.136

Thalamus L 4.0855 -6 -16 14 4.088

Thalamus L 3.9824 -12 -18 8 11.408

Brainstem/Cerebellum

Cerebellum_10 R 3.8905 28 -38 -46 3.608

Cerebellum_6 R 4.127 32 -48 -24 0.752

Cerebellum_6 R 4.4044 32 -50 -30 2.928

Cerebellum_Crus1 L 3.9187 -36 -54 -36 4.312

Cerebellum_6 R 4.6328 26 -54 -24 0.872

Cerebellum_6 L 4.1907 -28 -54 -32 3.224

Cerebellum_6 R 4.3389 36 -56 -28 1.952

Cerebellum_4_5 R 3.8291 14 -56 -20 1.944

Cerebellum_Crus1 R 4.1218 38 -58 -34 3.592

Cerebellum_4_5 R 4.2286 10 -58 -16 0.936

Cerebellum_6 R 5.0563 28 -62 -22 3.888

Vermis_6 4.2229 4 -64 -24 4.064

Cerebellum_8 R 4.4853 22 -66 -52 6.88

Cerebellum_6 L 3.9024 -24 -66 -28 6.432

Cerebellum_6 R 4.8086 30 -68 -20 1.264

Cerebellum_6 R 4.701 24 -70 -22 1.984

Cerebellum_8 R 3.9206 16 -72 -44 0.384

Cerebellum_Crus2 R 4.0459 8 -74 -36 4.6

R, right; L, left. Coordinates are in Montreal Neurological Institute (MNI) space.

**S2b Table. Group average results during the ictal phase.** Brain activations in ictal (migraine) state in response to noxious heat applied to the dorsum of the hand.

**Regions Side z-stat Peak MNI coordinates**

**X(mm) Y(mm) Z(mm) Vol(cm^3^)**

Frontal

Superior_Orbital R 4.5305 34 54 10 3.536

Superior R 3.9114 12 34 54 2.384

Supp_Motor_Area L 4.2008 -2 14 54 4.488

Middle L 3.9141 -42 12 36 1.784

Supp_Motor_Area R 4.3996 8 10 50 1.304

Supp_Motor_Area R 3.9583 12 10 60 1.464

Supp_Motor_Area R 4.2068 8 8 70 6.032

Supp_Motor_Area L 4.7348 -2 8 44 2.384

Precentral L 4.564 -52 8 36 4.944

Inferior_Operculum R 4.6548 56 8 14 1.904

Supp_Motor_Area L 4.0272 -6 4 64 5.76

Middle L 4.0676 -28 4 62 7.384

Inferior_Operculum R 4.1258 46 2 22 6.976

Parietal

SupraMarginal R 4.1631 64 -24 32 3.624

SupraMarginal R 4.0255 52 -30 26 1.688

Inferior L 4.0272 -58 -30 44 2.344

SupraMarginal R 3.9202 56 -32 48 1.32

Inferior L 4.2575 -52 -32 48 3.968

SupraMarginal R 4.0329 54 -34 38 2.6

Postcentral L 4.0089 -42 -34 60 2.424

SupraMarginal R 4.0414 60 -36 34 0.784

Inferior R 4.2068 54 -38 48 1.064

Inferior R 4.1974 52 -38 54 1.512

Inferior L 4.5837 -38 -38 48 2.648

Inferior L 4.1808 -58 -38 46 0.688

Inferior R 4.4411 42 -40 44 3.536

Inferior L 4.3429 -40 -40 44 3.6

Inferior L 4.057 -56 -40 42 4.736

SupraMarginal R 3.9716 64 -42 26 0.448

SupraMarginal R 4.6414 62 -44 32 1.256

Inferior R 4.3342 42 -50 52 3.264

Inferior R 4.0187 48 -50 42 1.624

Occipital

Rolandic_Operculum R 4.4578 58 8 10 1.456

Rolandic_Operculum L 4.2433 -54 -2 4 3.272

Temporal

Superior L 4.2547 -52 2 0 2.744

Superior R 3.9352 64 -28 20 0.568

Superior R 4.0384 60 -30 20 1.216

Cingulum

Anterior L 4.1349 -6 26 24 3.472

Anterior L 3.9723 -6 20 26 3.112

Middle R 4.8118 8 12 40 2.888

Middle L 4.3377 -8 10 42 1.224

Middle R 4.4993 2 6 36 1.096

Insula

Anterior R 4.0423 34 18 -8 1.504

Anterior R 5.2598 38 12 -2 15.936

Anterior L 4.2083 -44 0 0 1.76

Anterior L 4.1858 -40 0 0 3.232

Posterior R 3.9829 40 -8 -8 1.888

Sub-Cortical

Caudate L 3.9556 -14 12 8 1.04

Putamen R 4.5868 22 8 2 7.056

Putamen L 4.1245 -22 2 -10 3.936

Putamen L 4.2648 -22 0 8 3.224

Amygdala R 4.0667 24 0 -12 2.992

Caudate R 4.3962 16 -4 16 1.672

Thalamus R 4.6413 10 -6 4 2.688

Thalamus L 4.1725 -12 -10 14 1.672

Putamen L 3.9925 -24 -10 8 2.672

Thalamus R 5.0102 14 -14 8 4.976

Thalamus R 4.3488 8 -14 0 0.512

Thalamus L 4.3032 -6 -14 10 1.752

Thalamus L 4.757 -14 -16 4 4.544

Thalamus R 3.9382 8 -18 -2 0.328

Thalamus R 4.078 8 -22 0 1.544

Brainstem/Cerebellum

Cerebellum_4_5 R 4.4068 20 -50 -22 5.008

Cerebellum_Crus2 L 3.9757 -40 -56 -40 5.128

Vermis_6 3.8976 4 -60 -20 8.88

Cerebellum_Crus2 L 4.0011 -38 -60 -40 2.296

Cerebellum_Crus1 L 3.9685 -40 -60 -32 2.96

Cerebellum_Crus1 R 3.9967 40 -62 -32 2.184

Cerebellum_6 R 4.0413 32 -64 -28 1.08

Cerebellum_6 R 4.149 28 -66 -24 2.616

Cerebellum_Crus1 R 4.1455 34 -68 -28 1.744

Cerebellum_6 L 4.2511 -26 -68 -18 1.744

Cerebellum_6 L 4.2323 -26 -68 -22 0.776

Cerebellum_Crus1 L 5.1212 -26 -72 -28 5.192

Cerebellum_7b R 4.1311 6 -74 -40 7.064

Pons R 3.9703 4 -26 -22 3.144

R, right; L, left. Coordinates are in Montreal Neurological Institute (MNI) space.
